# Liposome‐Encapsulated Melatonin Mitigates Amoxicillin‐Induced Neurotoxicity in a Zebrafish

**DOI:** 10.1111/jcmm.70969

**Published:** 2025-11-26

**Authors:** Ranjith Balakrishnan, Rajasekaran Subbarayan, Rupendra Shrestha, Dhasarathdev Srinivasan, Reena Shrestha, Ankush Chauhan, Dinesh Murugan Girija

**Affiliations:** ^1^ Centre for Advanced Biotherapeutics and Regenerative Medicine, Faculty of Research, Chettinad Hospital and Research Institute Chettinad Academy of Research and Education Kelambakkam Tamil Nadu India; ^2^ Centre for Herbal Pharmacology and Environmental Sustainability, Chettinad Hospital and Research Institute Chettinad Academy of Research and Education Kelambakkam Tamil Nadu India; ^3^ Department of Natural and Applied Sciences Nexus Institute of Research and Innovation (NIRI) Lalitpur Nepal; ^4^ Department of Internal Medicine Berkshire Medical Center Pittsfield Massachusetts USA; ^5^ Vopec Pharmaceuticals Pvt Limited, Research and Development Division Chennai Tamil Nadu India

**Keywords:** amoxicillin, liposomes, melatonin, nanoformulation, neurotoxicity, zebrafish

## Abstract

Amoxicillin (Amx), a β‐Lactam antibiotic frequently used to treat bacterial infections, has been linked to neurological effects, including anxiety, hyperactivity, ambiguity, seizures, and behavioural changes. We examined the neurotoxic effects of Amx in zebrafish and investigated the potential of liposome‐encapsulated melatonin (L‐Mel) as a therapeutic intervention. Computational studies have indicated that Amx and Mel interact with GABA receptors, suggesting the potential of L‐Mel in mitigating Amx‐induced neurological changes. Our findings demonstrated that the nanoformulated L‐Mel showed reduced toxicity in zebrafish larvae. Administration of L‐Mel to Amx‐affected zebrafish brain tissue significantly lowered the levels of reactive oxygen species, antioxidants (catalase, superoxide dismutase, and nitric oxide), and proinflammatory cytokines (TNF‐α, IL‐1β, and NF‐kB), based on the fixed EC‐50. Behavioural assessments revealed that L‐Mel treatment notably enhanced the immobility time and swimming performance, improving the movement abilities of zebrafish with Amx‐induced neuroinflammation. Moreover, the GABA/glutamate levels in the neural tissues exhibited significant recovery in the L‐Mel group. Gene and protein analysis showed substantial increases in BDNF, CREBBP, ASCL, NF‐κB and GABA‐A R γ2 in L‐Mel treated subjects. Histopathological evaluation revealed that L‐Mel treatment markedly attenuated Amx‐induced neurotoxicity, as evidenced by reduced neuronal degeneration and necrosis in the brain tissue, indicating a pronounced neuroprotective effect. In conclusion, our research suggests that L‐Mel is a promising therapeutic agent for mitigating Amx‐induced neurotoxicity.

## 1 Introduction

Antibiotics are extensively used worldwide; however, their exact global consumption remains unknown owing to incomplete national statistics [1]. Antibiotics enter aquatic environments through sewage, hospital waste, industrial effluents, and improper disposal, leading to detectable residues in surface and ground water [2, 3]. Amoxicillin (Amx), a widely prescribed β‐lactam antibiotic for bacterial infections caused by Streptococcus, is frequently detected in aquatic systems at concentrations ranging from nanograms to micrograms per litre in urban sewage, wastewater effluents, and rivers [4, 5, 6]. Its high usage, incomplete metabolism, and persistence make Amx a priority pollutant of concern, with potential risks to nontarget aquatic species through continuous low‐level exposure [7]. The occurrence of Amx in environmental samples worldwide is listed in Table 1.

**TABLE 1:** Occurrence of amoxicillin in environmental samples worldwide.

Drug	Country	Detected in	Concentration	References
Amoxicillin	Australia	Raw sewage	280 ng/L	[8]
	China	Urban sewage	3380 ng/L	[9]
	India	Aquifers, Surface	0.18 ± 0.16 μg/L, 0.18 ± 0.20 μg/L	[10]
	Italy	Effluent from sewage treatment plants	120 ng/L	[8]
	United Kingdom	Surface water	120 ng/L	[11]

Abbreviations: ng/L, nanogram/litre; μg/L, microgram/litre.

Moreover, their possible neurotoxic effects, particularly on the brain, have not been thoroughly investigated yet. Therapeutic interventions to mitigate Amx‐induced neurotoxicity are limited [12]. Antioxidant‐based interventions aim to neutralise reactive oxygen species (ROS) and reduce oxidative stress, a major pathway in contaminant‐induced cellular injury. Chelation therapy is widely employed for the removal of toxic metals, such as lead, mercury, and arsenic, thereby minimising their bioavailability and systemic effects. Bioremediation‐assisted approaches, including probiotic supplementation and enzymatic degradation, have shown promise in reducing the contaminant burden through microbial metabolism. Additionally, nanotechnology‐driven drug delivery systems are being explored to achieve targeted detoxification with minimal off‐target effects. Collectively, these strategies offer complementary mechanisms for mitigating the adverse health effects of contaminant exposure and support the development of integrative detoxification protocols. Our study investigated the neuroprotective efficacy of nanoformulated melatonin (Mel) in mitigating Amx‐induced neurotoxicity, employing zebrafish ( *Danio rerio* ) as a preclinical model owing to their high genetic, neurochemical, and physiological similarity to mammals, as well as their well‐characterised nervous system, and suitability for neurobehavioural and toxicological assessments. Melatonin, an indoleamine produced in the pineal gland, influences biological processes through its interactions with G protein‐coupled melatonin receptors. It is essential for neuroprotection, partly because of its role in increasing gamma‐aminobutyric acid (GABA) levels in the central nervous system [13, 14].

Melatonin levels fall dramatically beyond the age of 50 years, leading to the onset of neurological and developmental problems in both men and women [15]. Exogenously administered synthetic melatonin is used to treat sleep disorders caused by circadian rhythm abnormalities, such as shift work, jet lag, or insomnia [16, 17, 18]. In addition to its function in the regulation of circadian rhythms, melatonin serves as an effective free radical scavenger that exhibits both anti‐inflammatory and antioxidative characteristics. It can neutralise ROS that arise from external stressors, including ultraviolet radiation, alcohol, and environmental toxins [19]. A previous study indicated that melatonin enhanced cognitive function, diminished neuroinflammation, and restored the integrity of the blood–brain barrier (BBB) in murine models of neuroinflammation [20].

However, the rapid systemic elimination of melatonin, coupled with its restricted bioavailability after oral or intravenous delivery, poses a challenge to its therapeutic effectiveness. To overcome these limitations, we propose encapsulating melatonin within a 10,12‐pentacosadiynoic acid (PCDA) and 1,2‐dimyristoyl‐sn‐glycero‐3‐phosphocholine (DMPC)‐based liposomal system to enhance stability, extend release, and improve neuroprotective effects. The initial hypothesis of this study focused on characterising PCDA‐DMPC liposomal encapsulation of melatonin (L‐Mel). Its efficacy in vitro was evaluated by measuring the IC50 value and examining its effects on mitochondrial function [21]. Furthermore, molecular docking and computational simulations were used to investigate the binding affinities of Amx and Mel to the GABA receptor, clarifying their molecular interactions and their effects on neurotransmitter signalling. Western blot analysis was performed to assess the expression levels of GABA proteins in the signalling pathways affected by Amx.

The second hypothesis focuses on assessing the safety and toxicity of L‐Mel in vivo, using zebrafish as a model organism. The EC50 concentration will be determined to establish a safe and effective dose while assessing developmental abnormalities linked to L‐Mel exposure. This study assessed the possible beneficial properties of L‐Mel against Amx‐induced neurotoxicity using behavioural assays, markers of oxidative stress, cholinergic function, protein and gene expression analysis, and histopathological examination. Furthermore, high‐performance liquid chromatography (HPLC) was used to measure the levels of GABA and glutamine receptors, supporting the findings of the computational analysis. This study sought to demonstrate that L‐Mel can reduce neurotoxicity induced by Amx in zebrafish, presenting a new therapeutic strategy to alleviate the neurotoxic side effects associated with the use of antibiotics.

## 2 Materials and Methods

### 2.1 Materials

High‐quality reagents and consumables were used in this study. Amoxicillin (CAS: 26787‐78‐0) and Melatonin (CAS: 73‐31‐4) were purchased from Sigma‐Aldrich (St. Louis, MO, USA). The Rat Cortical Neuron cell line (SKU: 10RA‐032) and Rat Cortical Neuron Maintenance Medium (SKU: MD‐0107B) were obtained from iXCells Biotechnologies (San Diego, CA, USA). JC‐1 dye (2.5 mM) and Rhodamine B (CAS: 81‐88‐9) were sourced from Sigma‐Aldrich (St. Louis, MO, USA). ELISA kits for TNF‐α, IL‐1β, and NF‐κB were purchased from MyBioSource (San Diego, CA, USA). The Verso cDNA Synthesis Kit was purchased from Thermo Fisher Scientific (St. Louis, MO, USA). Primers for gene expression analysis were custom‐synthesised by Eurofins Genomics (Bangalore, India). Chemicals and consumables for protein analysis, including buffers and electrophoresis‐grade reagents, were purchased from Lonza (Basel, Switzerland).

### 2.2 Computational Analysis

#### 2.2.1 Active Site Prediction

An automated pocket discovery technique called Dog‐site Scorer was used to predict the active sites. In addition, the drug potential of a specific site was evaluated based on its physicochemical and geometric characteristics (https://proteins.plus/) [22]. The figure was created using PyMOL software. BIOVIA Discovery Studio was used to conduct an in‐depth analysis of the molecular properties and chemical structure. This powerful tool allowed us to perform virtual screening and accurately predict the target areas of the molecules. In addition, it provided detailed 2D and 3D structures for further examinations [23].

#### 2.2.2 Molecular Docking

Our structure‐based drug design approach revealed significant interactions between proteins and low‐energy ligands. We utilised AutoDock to analyze the binding affinity and ligand efficacy of the GABA receptors (Amx and Mel). After performing the necessary computations, a final energy evaluation was conducted using the docking scores. Docking was performed using AutoDock Vina. A grid box was created using the AutoDock tool, and docking was performed within the specified region. Kollman charges (4.752) of hydrogen atoms were integrated into the protein, whereas Gasteiger charges and hydrogen atoms were applied to the ligand. The grid box was generated using Auto Grid 4, with box dimensions of X = −43.529, Y = 8.895, and Z = −11.826 (GABA/Amx). Using AutoDock Vina, a set of nine poses was generated using the receptor and ligand files, as well as a configuration file that contained grid box properties. An observation was made regarding the interaction between the docking pose and the residues present in the active site.

### 2.3 Synthesis, Characterisation and Encapsulation Efficiency of PCDA‐DMPC Liposome

Melatonin was synthesised into PCDA and DMPC assemblies using a previously established protocol to ensure the validity and reliability of our method [24]. First, a blend of PCDA and DMPC was prepared by dissolving them in trichloromethane in a round‐bottom flask at a proportion of 4:1 M. The mixture was then combined with melatonin in methanol. An immaculate buffy coat layer was prepared by evaporating the solvent at 45°C using a rotary evaporator. The final layer obtained was washed with 1× PBS (pH 7.2) and subjected to sonication for 30 min at 45°C–50°C. The final mixture was filtered through (0.4 μm) and centrifuged at 402 *g* for 5 min to collect the pellets (L‐Mel). The samples were then incubated at 4°C [21]. The L‐Mel characterisation was performed using a UV–Vis spectrophotometer (Cary 5000 UV‐SPEC) in the spectral range of 300–700 nm. The particle size distribution was measured using a Nanopartica SZ‐100 instrument. The Fourier transform infrared (FTIR) and attenuated total reflectance (ATR) spectra of the samples in both solid and liquid states were recorded at room temperature using an FTIR spectrometer (Bruker, ALPHA‐T model) equipped with an ATR accessory and operated with OPUS 6.5 software. For solid samples, the potassium bromide (KBr) pellet method was employed, in which the finely ground sample was mixed with spectroscopic grade KBr and pressed into a transparent pellet prior to measurement. Liquid samples were directly placed on the ATR crystal without further treatment. All spectra were collected in the range of 500–3000 cm^−1^ with a spectral resolution of 4.0 cm^−1^. X‐ray photoelectron spectroscopy (XPS) was performed using a Thermo Fisher Scientific K‐Alpha spectrometer (USA) to analyse the surface chemical modifications of melatonin encapsulated within liposomal formulations. Optical microscopic images were captured using a high‐quality Leica DM6 microscope at 40× magnification. Ultrastructural images were acquired using a transmission electron microscope (TEM; JEM‐2100Plus) with a 200 nm range.

### 2.4 Drug Release Profile of Liposome‐Encapsulated Melatonin (L‐Mel)

Dialysis was performed to analyze the kinetics of melatonin release from L‐Mel. A total suspension of L‐Mel (3 mL) was placed into a dialysis bag and submerged in a solution of 5% methanol/10 mL 1XPBS with a pH of 7.2 at 38°C, and a 2 mL sample of the release medium was collected at 6 h intervals and replaced with a similar quantity of the original PBS solution to assess the amount of melatonin released. The emission spectra from 300 to 500 nm were measured using a spectrophotometer (Cary 5000 UV‐SPEC), and a plot of the emission intensity against time was created. This emission intensity was used to determine the amount of melatonin released from L‐Mel compared to that released from Mel [21].

### 2.5 Neuron Cell Culture

Rat cortical neurons were cultured in a controlled environment with a conditioned atmosphere, 5% CO_2_, and a temperature of 37°C in the prescribed medium supplemented with growth factors, 1% penicillin–streptomycin, and 2.5% FBS. The cells were grown in a monolayer until they attained 80%–90% confluency. Cells were counted using a haemocytometer at a concentration of 1 × 10^5^ cells.

### 2.6 Cell Viability Assay of Neuron Cells Treated With L‐Mel

Cells were cultured overnight, and the MTT assay was performed according to a standard protocol. For the Mel experiment, the cells were treated with 1, 4, 6, 16, 28, and 36 mM Mel. Similarly, for the L‐Mel sets, the cells were treated with 0, 1.56, 3.12, 6.25, 12.5, 25, 50, and 100 μg/mL L‐Mel. Subsequently, both Mel‐ and L‐Mel‐treated cultures were incubated for 48 h. After incubation, 20 μL of MTT solution was added to each well and mixed carefully. After 4 h, the aqueous phase was removed, and 100 μL of DMSO was added to disperse the precipitate. The absorbance at 570 nm was measured using a Quant ELISA plate reader (Bio‐Tek Instruments, USA) to determine the cell survival. Cytotoxicity (IC50) was determined by measuring the percentage of cell death after drug treatment [25]. Cell viability was determined using the following formula:

%Cellviability=absorbancesample/absorbancecontrol×100

### 2.7 Mitochondrial Membrane Potential Study on Neuron Cells Treated With L‐Mel

JC‐1 staining was performed to study the mitochondrial membrane potential (MMP) of L‐Mel‐treated cells. Briefly, the cultures were divided into different treatment groups at the following final concentrations: 25 μM Amx, 8 mM Amx/Mel, and 5 μg/mL Amx/L‐Mel. The cells were incubated in a CO_2_ incubator at 37°C for 24 h. After incubation, the cells were exposed to JC‐1 dye at a concentration of 2.5 μM (dissolved in 100 mM Tris, pH 8.2), gently agitated, and kept in the dark for 15 min. The supernatant was then removed, and the cells were examined using a Leica SPE confocal microscope. Samples at 488 and 550 nm demonstrated excitation and detection of both green and red fluorescence, respectively [26, 27].

### 2.8 Zebrafish Husbandry and Embryo Collection

Zebrafish were procured from a Beryl Aqua fish farm in Chennai, India, and maintained in a laboratory with proper husbandry facilities. Thirty adult zebrafish (~50% male/female), aged 6–8 months, with an average length of 3–4 cm and a body weight of approximately 0.3–0.5 g, were used in this study. The fish were kept in a container with a water volume of 2 fish/L for 30 days before testing. During this time, they were kept in dechlorinated tap water with a pH range of 7.1–7.5 at a temperature of 25°C ± 3°C. The fish were exposed to a consistent daily cycle of 12 h of daylight and 12 h of darkness to ensure that the oxygen saturation remained above 80%. They were also fed twice a day according to the ‘Test Guideline No. 203 Fish, Acute Toxicity Testing (OECD 2013)’. After acclimatisation, they were separated by sex and kept in separate tanks for a week before breeding. Random mating was applied to produce embryos, and four males and eight females were placed in a mating container, as previously reported [28]. Fertilised embryos were washed and raised in E3 medium. Following a previous study, embryos were exposed to 100 mg/L Amx for 96 h (starting at day 1, 09:00 AM, and ending at day 4, 09:50 AM) [7]. The 100 mg/L Amx concentration showed significant but nonlethal effects in both zebrafish embryos and adults, making it suitable for evaluating sublethal developmental and physiological responses. This concentration also supports reproducibility and allows for meaningful comparisons with the existing toxicological data [7]. All experiments were conducted according to the CPCSEA Guidelines for Fish Experimentation (2021) and were approved by the Institutional Animal Ethics Committee (IAEC) (IAEC 3/Proposal:86/A. Lr:62).

### 2.9 Development and Toxicity Study in Zebrafish Exposed to L‐Mel

The effective concentration of L‐Mel was determined by exposing the zebrafish embryos to different concentrations of L‐Mel (0, 2, 4, 6, and 8 mg/L) for 96 h. Embryos (4 days post fertilisation) were then observed, and images were captured to identify dead and live embryos. For the efficacy analysis, experiments were performed on larvae (*n* = 10) and adults (*n* = 10) [29]. The sample size of 10 zebrafish per group was determined based on established methodologies from previous studies that used zebrafish as a neurotoxicity model. Zebrafish offer robust and reproducible neurobehavioural endpoints, with similar sample sizes that provide adequate statistical power for detecting significant treatment‐related effects [29]. The effect of L‐Mel on Amx‐induced toxicity was evaluated by exposing zebrafish embryos for 96 h to three different treatment groups: Amx dissolved in water (Amx/H_2_O), Amx co‐administered with free melatonin (Amx/Mel), and Amx co‐administered with L‐Mel (Amx/L‐Mel). Each exposure was conducted under standard zebrafish embryo‐rearing conditions, and developmental, behavioural, and biochemical parameters were monitored to assess treatment‐specific effects. The treatment dose was scheduled for 4 days, and in the case of adult zebrafish, the dosage was administered orally using a micropipette; this choice is significant as it represents the standard exposure times for toxicity studies using zebrafish, following the established guidelines of the OECD (Test number 203, 201 guidelines 3) [30].

### 2.10 Behavioural Analysis

Naïve larvae (*n* = 10 per group) at 6 days post‐fertilisation were used in this experiment. Each group of larvae (Con, Amx, Amx/Mel, and Amx/L‐Mel) was carefully placed in a Petri dish containing 2 mL of E3 medium. The Petri dish was positioned on a steady tripod platform, and larval movement was recorded using a mobile phone. The recordings were conducted hourly, with each recording lasting 6 min for a total duration of 12 h. ANY‐maze software was used to analyse locomotion function, track swimming movements, and accurately determine the locomotion of the larvae [31]. Adult zebrafish locomotor activity was further assessed using a Python (v3.12.5) ‐based pipeline (OpenCV, Matplotlib) to generate track plots and heatmaps, enabling group‐wise comparisons of treatment‐specific effects on locomotor and exploratory behaviour.

### 2.11 Enzyme‐Linked Immunosorbent Assay (ELISA)

ELISA BioTek Synergy HTX (BioTek Instruments, USA) was used to determine the levels of antioxidant enzymes and proinflammatory cytokines in larval samples. After 96 h of drug treatment, the larvae (*n* = 10) were dissected, and their tissues were homogenised using 500 μL of protein lysis buffer. The protein extract was dissolved in 500 μL ELISA buffer. The levels of CAT (#MBS705697), SOD (#MBS705758), NO (#MBS2540417), GTPx (#MBS024388), GST (#MBS109727), GSH (#MBS168487) were measured according to the manufacturer's instructions (My BioSource, USA). In addition, we estimated the levels of TNF‐α, IL‐1β, and NF‐κB (BioSource) in brain samples, following the manufacturer's guidelines. Furthermore, acetylcholinesterase (AChE) activity in adult zebrafish brain tissue was assessed using the Acetylcholinesterase Assay Kit (Colorimetric) (ab138871; Abcam, USA) according to the manufacturer's protocol.

### 2.12 Quantitative Polymerase Chain Reaction (qPCR)

Four specific genes were analysed for gene activity: BDNF, CREBBP, ASCL, and NF‐κB. Briefly, TRIzol reagent was used to extract RNA from the heads of treated larvae (*n* = 10), and the RNA quantity was determined using a Nanodrop 2000c (Thermo Fisher Scientific, USA) following the manufacturer's protocol. Next, reverse transcription of RNA to cDNA was performed using the ThermoFisher Verso cDNA Synthesis Kit [32]. The primers used are listed in Table 2. qPCR was conducted using SYBR Green PCR Master Mix (Applied Biosystems, UK) on a Quanta Studio 6. The fold change was determined by normalising the expression of the target genes to that of β‐actin. All qPCR reactions were performed in triplicates.

**TABLE 2:** qPCR primers.

Gene	GenBank accession no.	Forward primer	Reverse primer	References
BDNF	NM_131595	5′AACTCCAAAGGATCCGCTCA3′	5′GCAGCTCTCATGCAACTGA3′	[33]
CREBBP	NC_007133	5′CGAAAAGTGGAAGGGGACAT3′	5′TTCTCTTCCAGCTCTTTCTGG3′	[34]
ASCL	NC_007115	5′TGAGCGTTCGTAAAAGGAAACT3′	5′TGGCTCTTTGACACTCGGAC3′	[35]
NFκB	NC_007131	5′AGTCATGCCAGAGAGCGAAT3′	5′CAGAGCCGGATGTCATCATA3′	[36]
β‐Actin	NM_131031	5′CACAGATCATGTTCGAGACC3′	5′GGTCAGGATCTTCATCAGGT3′	[33]

### 2.13 IVIS Imaging

A live imaging system was used to assess Rhodamine‐tagged L‐Mel tracers using the IVIS Lumina LT series III (Caliper, MA, USA). Rhodamine B (0.2 m%) was dissolved in PBS along with the L‐Mel construct to a final volume of 20 μm/mL and shaken in the dark for 10 min. The adult fish (*n* = 10) treated with Amx were orally administered rhodamine‐tagged L‐Mel (10 μL) and placed in a tank with normal water for 4 h, after which the fish were taken from the tank, processed, and the fluorescence image was captured in two different time frames (4 and 12 h) [37, 38].

### 2.14 Histopathology Analysis

Brain, liver, and intestinal samples of adult fish (*n* = 10) were collected during the terminal procedure, fixed with 10% formalin, and processed for paraffinisation. All paraffin‐embedded tissues were sectioned into 5 μm sections and subjected to haematoxylin and eosin (H&E) staining [39].

### 2.15 High‐Performance Liquid Chromatographic Analysis (HPLC)

Brain tissues (adult zebrafish *n* = 10) were washed with cold saline solution (0.9%) and homogenised using 0.1 N hydrogen chloride in 70% ethanol. The samples were then centrifuged for 20 min at 1811 *g* at 25°C. The supernatants obtained were filtered using a 0.45 μm filter and diluted appropriately with the mobile phase for analysis using a Shimadzu LC‐20AT HPLC system (Shimadzu Corporation, Kyoto, Japan). The retention times (RT) were documented, and calibration curves were constructed using standard solutions to quantify the analytes in the samples. Chromatographic data were analysed by calculating the peak areas corresponding to GABA and glutamate [40].

### 2.16 Western Blot Analysis

Brain tissues from adult zebrafish (*n* = 10) were collected, and proteins were extracted using RIPA cell lysis buffer (SKU: AR0105‐100, Boster Bio, CA, USA) and maintained on ice for a minimum of 30 min. Lysates were centrifuged at 12,000 *g* for 10 min at 4°C, and the supernatant was transferred to a new tube. Protein concentration was determined using the bicinchoninic acid (BCA) assay. Equal amounts of protein were loaded per lane, separated by sodium dodecyl sulfate‐polyacrylamide gel electrophoresis, and transferred to PVDF membranes. Membranes were blocked with 10% bovine serum albumin (BSA) in 0.05% Tris‐buffered saline with Tween 20 (TBST) for 1 h at room temperature, followed by overnight incubation at 4°C with anti‐GABA‐A receptor gamma 2 antibody (R&D Systems, Catalog # PPS072). After three washes, the membranes were incubated with horseradish peroxidase (HRP)‐conjugated goat anti‐rabbit IgG (H + L) secondary antibody (Catalog #31460; Thermo Fisher Scientific) for 2 h at room temperature. Detection was performed using enhanced chemiluminescence (ECL) reagents (Pierce, Rockford, IL, USA) and visualised. Following immunodetection of GABA‐A, the membranes were stripped with stripping buffer and re‐probed with β‐actin antibody, which served as an internal loading control to confirm equal protein loading across all samples [41].

### 2.17 Statistical Analysis

Statistical analyses were performed on all experimental data. We used nonlinear regression analysis to determine the IC‐50 concentration. One‐way ANOVA with post hoc Bonferroni's multiple comparison test was performed for the molecular analysis. The significance level was determined by (*p < 0.05, **p < 0.01, and ***p < 0.001) using GraphPad Prism 10.0.

## 3 Results

### 3.1 Hydrogen Bonding of Amx and Mel With GABA Protein

**FIGURE 1:**
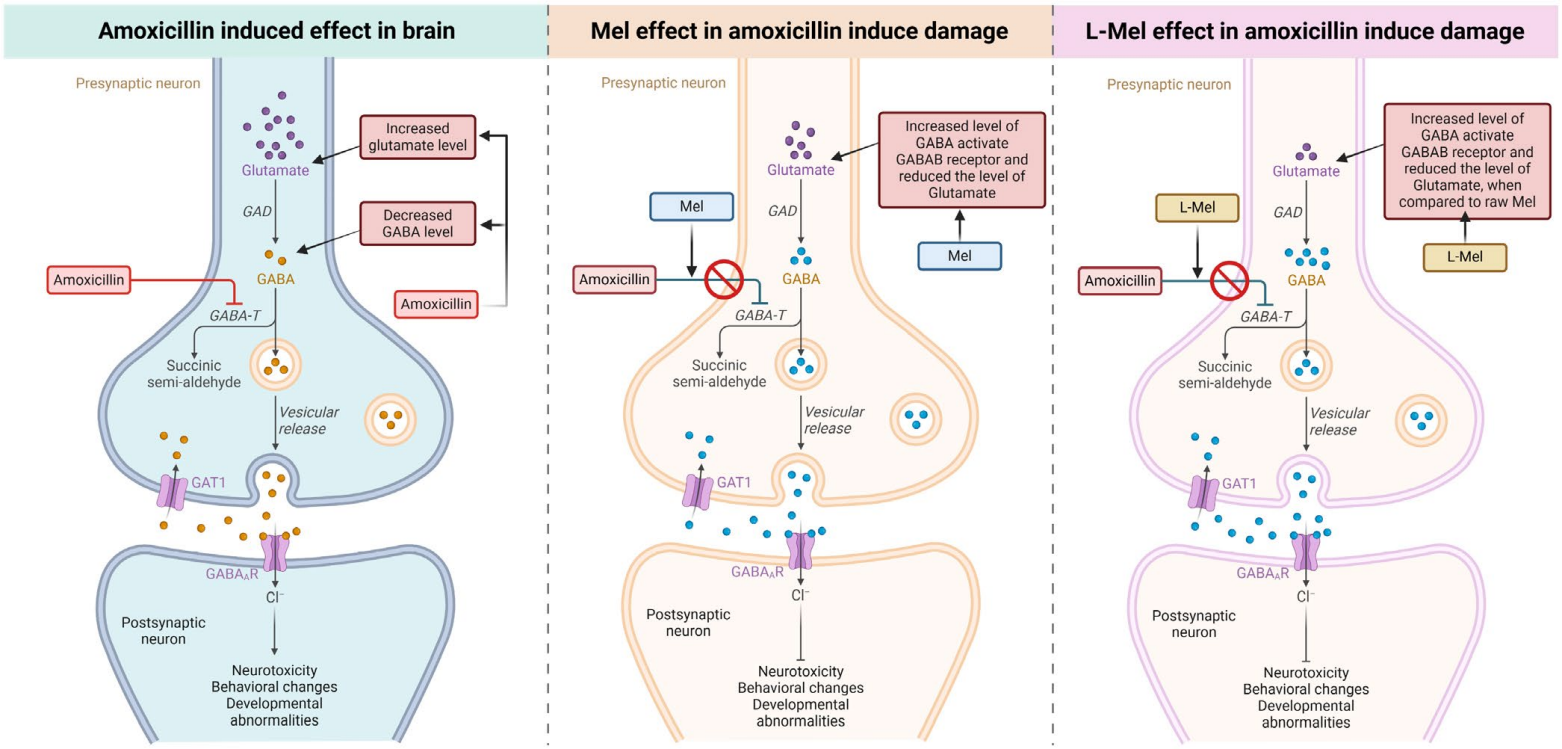
Illustration of the liposome‐encapsulated melatonin mechanism in ameliorating Amx‐mediated neurotoxicity.

**FIGURE 2:**
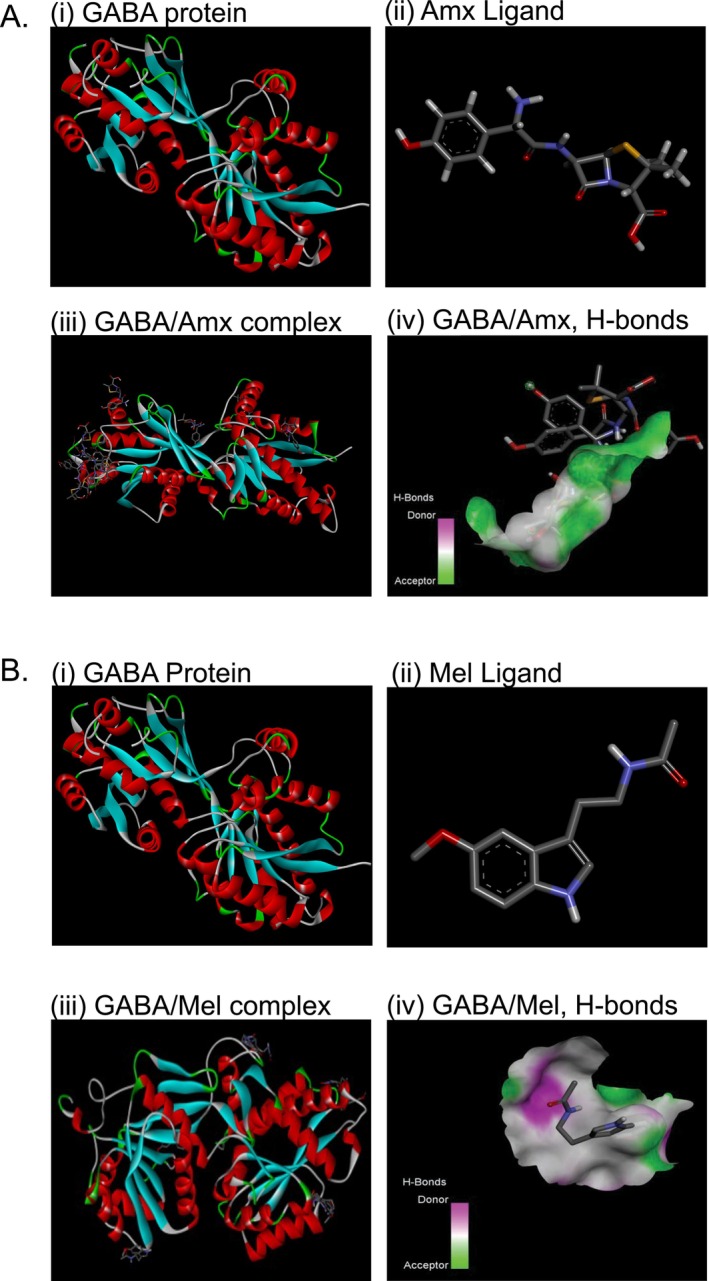
Molecular docking of Amx and Mel, showing binding affinity. 3D structure of the GABA protein (A and B, i). Undocked structures of Amx and Mel ligands (A and B, ii). The ribbon structure illustrates the 3D protein structures of GABA/Amx (A, iii) and GABA/Mel (B, iii). The H‐bonds demonstrate the strong binding affinity of the ligands Amx (A, iv) and Mel (B, iv). Amx, amoxicillin; Mel, melatonin.

**FIGURE 3:**
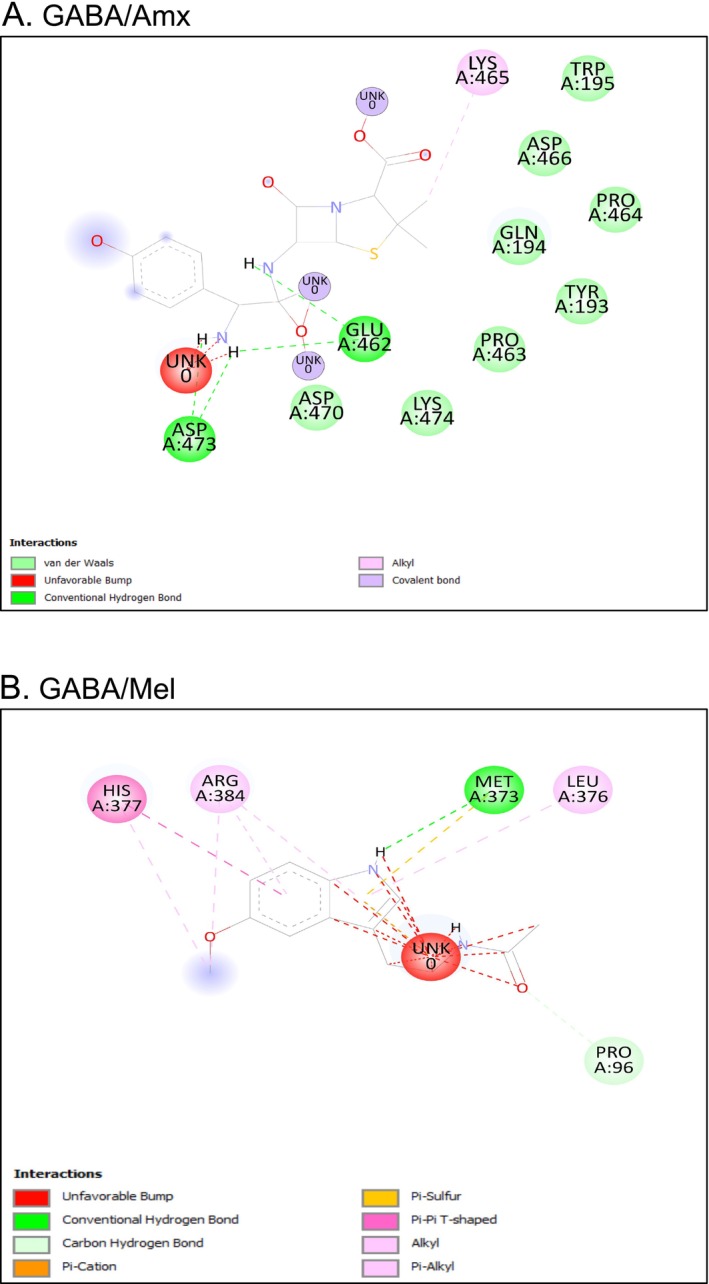
Docking of GABA with Amx (A) and Mel (B) ligands, revealing the interaction with important residues in the active site cleft.

The schematic representation demonstrates the interaction of melatonin and liposome‐encapsulated melatonin with the GABA receptor to ameliorate the effect of Amx‐mediated neurotoxicity (Figure 1). According to the schematic representation, our study demonstrated the successful docking of Amx (Figure 2A, i–iv) and Mel (Figure 2B, i–iv) (ligands) with their predicted receptor GABA. The 3D structure demonstrated the existence of a robust hydrogen bond between the ligand and its receptor molecules. Furthermore, SAS interactions and aromatic bonding between the protein (GABA) and ligands (Amx and Mel) were demonstrated (Figure S1). This study investigated 2D ligand interactions between Amx, Mel, and specific amino acid chains in the targeted protein molecules. Our study confirmed the interactions between Amx and Mel with specific protein sites. Furthermore, the Amx interaction was marked by a robust conventional hydrogen bond involving ASP (A:473) and GLU (A:462) bound to GABA (Figure 3A), and the Mel interaction was marked by a conventional hydrogen bond involving MET (A:373) bound to GABA (Figure 3B). Table 3 illustrates the pose that exhibits a stronger binding affinity, which has significant implications for understanding GABA receptors. Furthermore, Ramachandran plot analysis showed that a significant majority (80.0%) of the residues were situated in the favourable regions of both Amx and Mel while interacting with the GABA protein (Figure S2).

**TABLE 3:** Molecular docking of Amx and Mel showing binding affinity.

S. no.	Drug (ligand)	Protein	Binding affinity
1.	Amoxicillin (Amx)	GABA	−6.08
2.	Melatonin (Mel)	GABA	−5.40

### 3.2 Melatonin Encapsulation in Liposomes and Their Functional Characterisation

**FIGURE 4:**
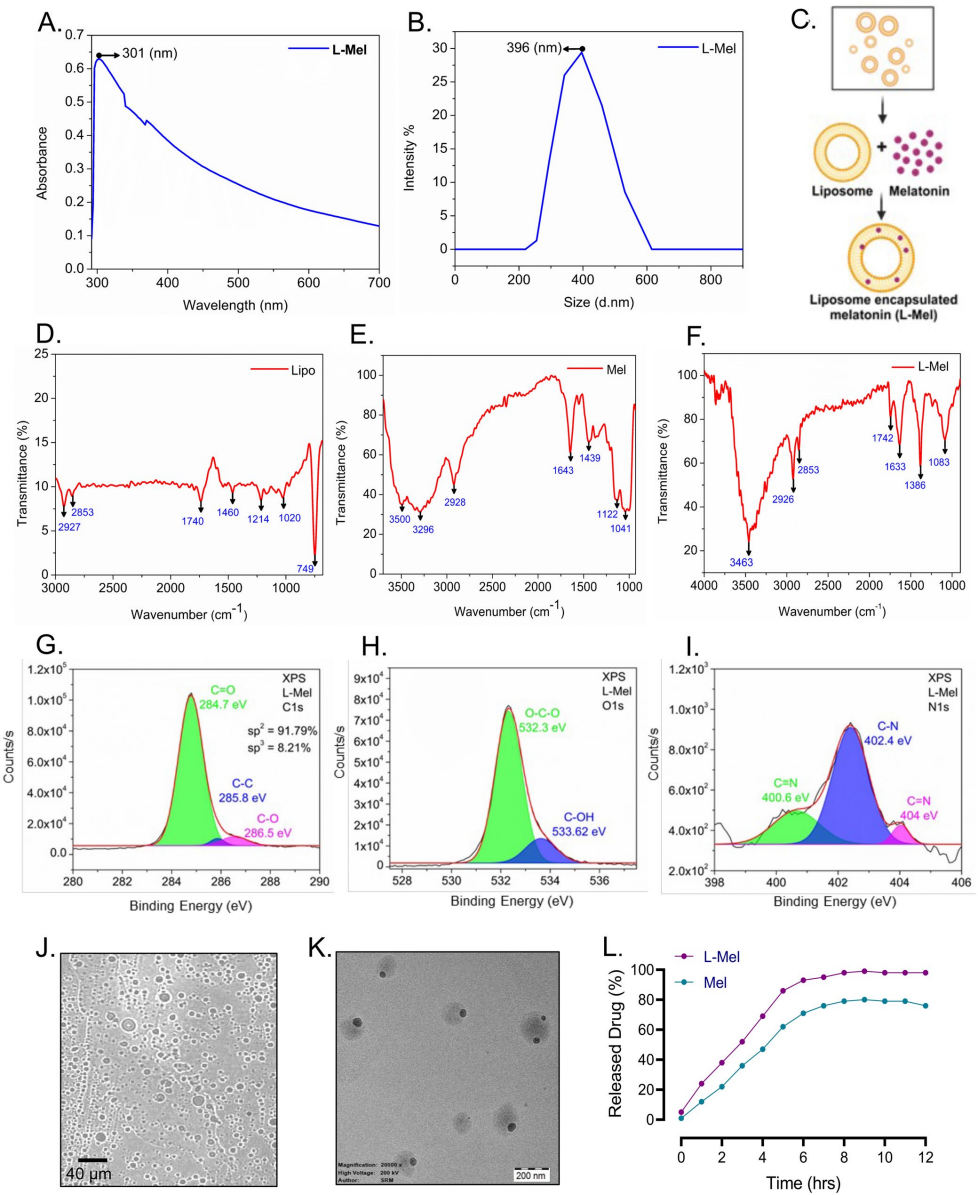
Characterisation of liposome‐encapsulated melatonin (L‐Mel). UV spectrum of L‐Mel (A). DLS of L‐Mel (B). Illustration of liposome encapsulation of melatonin (C). ATR‐FTIR spectra of liposomes (D), melatonin (E), and L‐Mel (F). High‐resolution XPS spectra of L‐Mel showing C1s with peaks for C=O (284.7 eV), C–C (285.8 eV), and C–O (286.5 eV) (G); O1s with O–C–O (532.3 eV) and C–OH (533.6 eV) (H); and N1s with C≡N (400.6 eV), C–N (402.4 eV), and C=N (404 eV) (I). These deconvoluted peaks confirm the bonding states of carbon, oxygen, and nitrogen in L‐Mel. Photomicrography of L‐Mel using light microscopy (J). TEM image of L‐Mel (K). Release Kinetics: The release of Mel and L‐Mel at different time intervals (L). Amx, amoxicillin, L‐Mel, liposome encapsulated melatonin.

Melatonin was encapsulated in a nanovesicular liposome composed of a polydiacetylene‐based polymer (PCDA) and phosphatidylcholine‐type lipid (DMPC) in a 4:1 M ratio. The hydrophobic tail and hydrophilic head groups of PCDA and DMPC organise in an elliptical two‐layer position in a fluid medium through self‐assembly in a professional manner. The nanoaggregates were removed by centrifugation and subjected to UV irradiation at 254 nm. Liposomal melatonin (L‐Mel) was analyzed using UV‐Spec, DLS, and FTIR. The electronic absorption spectrum of melatonin does not show absorption above 320 nm [42]. Based on our results with L‐Mel in PBS, the 5% methanol mixture showed a prominent peak at 301 nm, suggesting that melatonin was successfully encapsulated into the liposomes (Figure 4A). Previous studies have shown that liposomes with sizes ranging from 100 to 400 nm exhibit minimal interactions with plasma proteins, an extended lifespan in the bloodstream, and improved cellular absorption [43]. According to our DLS results, the average measurement for L‐Mel was 396 nm (Figure 4B). Based on our experiment, a schematic representation of liposome‐encapsulated melatonin is shown in (Figure 4C).

The ATR‐FTIR spectra of the liposome compositions are shown in (Figure 4D–F). The liposome exhibited peaks at 2927 and 2853 cm^−1^, indicating stretching between the CH_3_ and CH_2_ groups of the acyl chain, respectively. Additionally, a peak was observed at 1740 cm^−1^, suggesting the presence of C=O stretching, and another peak was observed at 1460 cm^−1^, indicating the scissoring of CH_2_ bending. Furthermore, a peak was observed at 1020 cm^−1^, indicating C–O stretching (Figure 4D) [44, 45]. Melatonin exhibited prominent peaks at distinct wavenumbers, suggesting the presence of functional groups. The peak at 3296 cm^−1^ corresponds to N–H bond stretching. The stretching of the C–H bonds caused another peak at 2928 cm^−1^. The spectral peak indicated amide I C=O stretching at 1643 cm^−1^. Other prominent peaks were C–H scissoring at 1439 cm^−1^, O–H bending at 1122 cm^−1^, and C–O stretching at 1041 cm^−1^ (Figure 4E) [44, 46, 47]. In contrast to liposomes and melatonin, L‐MEL exhibited several characteristic bands. These include the O–H stretching band at 3464 cm^−1^, acetyl chain peaks at 2926 and 2853 cm^−1^ corresponding to CH_3_ and CH_2_ stretching, the C=O stretching band at 1742 cm^−1^, the amide I C=O stretching band at 1633 cm^−1^, the CH_3_ stretching band at 1386 cm^−1^, and the C–O stretching band at 1083 cm^−1^ (Figure 4F). This result showed that Mel was perfectly encapsulated within the liposomes, which could enhance the stability and release time of melatonin in the case of antibiotic‐induced neurotoxicity.

High‐resolution X‐ray photoelectron spectroscopy (XPS) was employed to elucidate the chemical states of L‐Mel. The C1s spectrum was dominated by a peak at 284.7 eV, characteristic of C=O bonds, with minor contributions from C–C (285.8 eV) and C–O (286.5 eV), indicating that the carbon framework was primarily sp^2^‐hybridised (91.79%) with a small fraction of sp^3^ carbon (8.21%) (Figure 4G) [48, 49]. The O1s spectrum revealed two main components, assigned to O–C–O (532.3 eV) and C–OH (533.6 eV) functionalities, consistent with the presence of hydroxyl and carboxyl groups (Figure 4H) [48, 49]. The N1s spectrum exhibited peaks at 400.6, 402.4, and 404.0 eV, corresponding to C=N and C–N bonding environments, confirming the incorporation of nitrogen heteroatoms within the conjugated structure (Figure 4I) [48, 50]. Collectively, these results demonstrate that L‐Mel is enriched with sp^2^ carbon domains integrated with oxygen‐ and nitrogen‐containing functional groups, which underpins its chemical stability and functional reactivity. Thus, the current size of nanofromulations is ideal for drug delivery applications. Additionally, the polymerised liposomes were verified to have a spherical structure through photomicrography using light microscopy and TEM imaging (Figure 4J,K).

### 3.3 Encapsulation of Mel and Its Sustained Release From Liposome‐Mediated Carriers

In this study, the release kinetics of Mel and L‐Mel were determined in a simulated physiological environment for 12 h. According to the observations, there was a noticeable pattern in the amount of Mel released over time, with a steady increase up to 6 h. In contrast, L‐Mel required approximately 12 h to show a similar increase (Figure 4L). In L‐Mel, approximately 50% of the drug was released over 6 h, whereas in Mel, 100% of the drug was released within the same time frame. Nevertheless, the release profile reached equilibrium after 12 h. No notable changes were observed in the release profiles. The release process reached a stable point at this late stage. The results clearly indicate that the liposomes created can serve as reliable carriers for the regulated and sustained diffusion of melatonin.

### 3.4 Effect of L‐Mel on Neuron Cells Toxicity and Mitochondrial Membrane Potential

**FIGURE 5:**
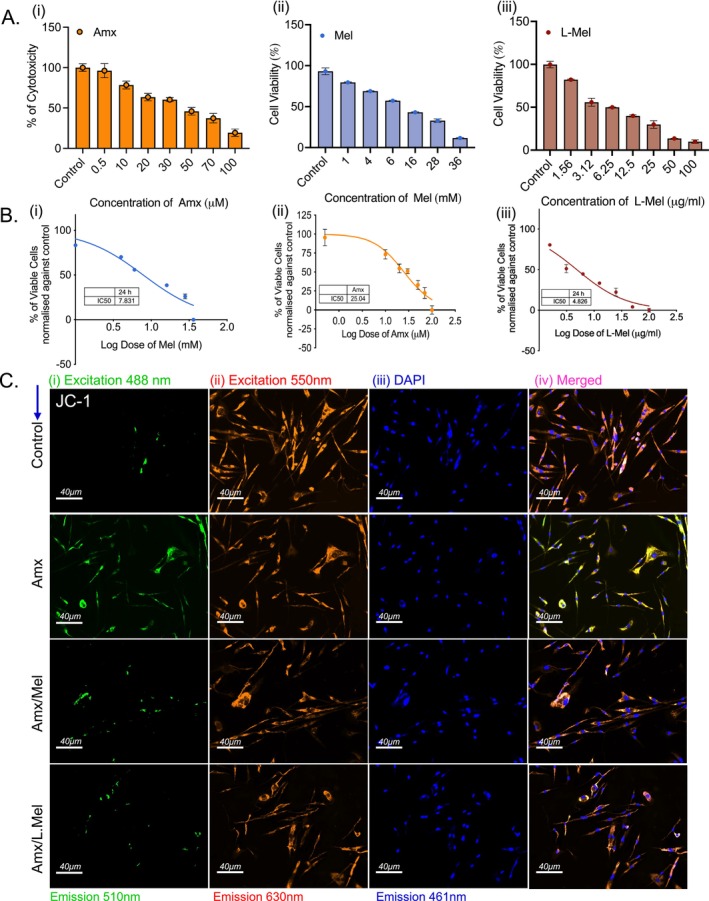
MTT assay illustrating the percentage of cytotoxicity. A non‐linear regression was conducted to determine the IC‐50 (A‐i, ii, and iii), the inhibitory concentration that shows 50% of cell death (B‐i, ii, and iii). Assessment of mitochondrial membrane potential by JC‐1 staining confirmed the control and treatment groups, and counterstaining of the nucleus with DAPI and merged images showed the co‐localization of the nucleus (C‐i, ii, iii, and iv). Images were obtained at 40× magnification with a 40 μm scale bar.

Cell viability was assessed using the MTT assay, which revealed that Amx, Mel, and L‐Mel exhibited inhibitory effects with an IC50 value of approximately 50%. After a 24‐h incubation period, cell viability decreased in a dose‐dependent manner (Figure 5A, i–iii). Furthermore, nonlinear regression analysis was conducted to quantify the proportion of viable and nonviable cells, determine the accurate IC50 value, and fix the concentration for further experimental analysis. Based on the MTT assay, the concentrations of Amx (25.04 μM), Mel (7.8 mM), and L‐Mel (4.82 μg/mL) are shown in (Figure 5B, i–iii). The effect of ROS on mitochondria was analysed using JC‐1 staining. JC‐1 is a fluorescent monomer that exhibits fluorescence under low membrane potential conditions, specifically in apoptotic cells. In these cells, JC‐1 binds to the cytoplasm and produces green fluorescence. In contrast, JC‐1 dye forms aggregates within the mitochondria at higher potentials in healthy cells, resulting in red fluorescence emission. Untreated control cells had a higher percentage of healthy cells with red fluorescence at 550 nm excitation, suggesting minimal membrane potential damage. This was confirmed by the absence of green fluorescence at 488 nm. In contrast, the Amx‐treated group showed a significant increase in the number of apoptotic cells, characterised by elevated green fluorescence at 488 nm. Notably, the L‐Mel treatment group showed a reduction in apoptotic cells, as evidenced by a decrease in green fluorescence (488 nm) compared to the Amx and Mel groups (Figure 5C, i–iv). These findings demonstrate that encapsulated L‐Mel is more stable and efficient than free Mel in preventing antibiotic‐induced neuronal damage.

### 3.5 Effect of L‐Mel on Zebrafish Development and Locomotor Activity

**FIGURE 6:**
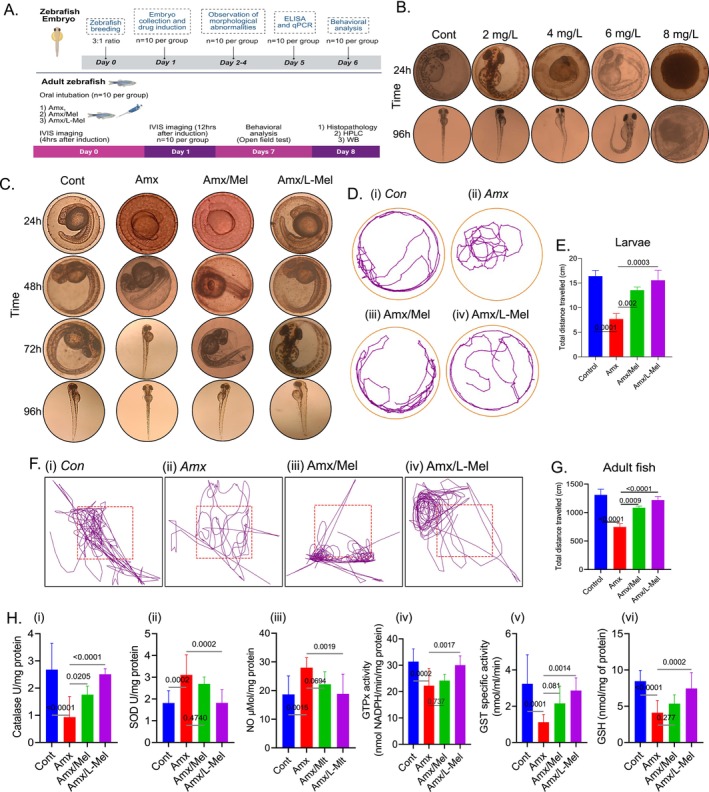
Schematic representation of the experimental design in zebrafish embryos and adults, including drug administration, imaging, behavioural assessment, and subsequent molecular and histopathological analyses (A). Impact of L‐Mel on zebrafish embryos (*n* = 10 per group) (toxicity test) (B). Morphological abnormalities caused by Amx exposure with and without L‐Mel (C). Locomotory behaviour of zebrafish larvae (*n* = 10 per group) was assessed using ANY‐maze software (D). Graphical representation of the total distance covered by larvae (*n* = 10 per group) (E). Representative track plots illustrating the locomotor activity of adult zebrafish in the open field test, generated using Python (v3.12.5) (F). Graphical representation of the total distance covered by adult zebrafish (*n* = 10 per group) (G). Changes in antioxidant enzyme activity in prenatal zebrafish embryos (*n* = 10 per group) exposed to Amx with and without L‐Mel combination (H). Amx, amoxicillin; L‐Mel, liposome‐encapsulated melatonin. Statistical significance between the Amx‐treated and Amx/L‐Mel‐treated groups was set at *p < 0.05, **p < 0.01, ***p < 0.001. Amx, amoxicillin; L‐Mel, liposome‐encapsulated melatonin; Mel, melatonin. (*p < 0.05, **p < 0.01, and ***p < 0.001).

A schematic representation of the experimental design in zebrafish embryos and adults is shown in Figure 6A. To determine the concentration of L‐Mel, EC50 was determined by exposing fertilised zebrafish embryos to concentrations of 0, 2, 4, 6, 8 mg/L of L‐Mel for 96 h. Embryos were observed, and images were captured to detect alive‐hatched, dead‐hatched, alive‐non‐hatched, and dead‐non‐hatched embryos after exposure to L‐Mel (Figure 6B). Following this experiment, a sublethal dose of 2 mg/L L‐Mel was selected. Furthermore, developmental abnormalities were observed when zebrafish embryos were exposed to 100 mg/L Amx for 72 h, leading to premature hatching. The effect of L‐Mel (2 mg/L) on Amx‐treated embryos showed complete development in zebrafish embryos without any abnormalities (Figure 6C). Subsequently, the larvae obtained from the previous experiment were used to assess locomotor activity, and it was found that Amx negatively affected the locomotion of zebrafish embryos. This was evident from the decreased total distance covered during the 5‐min recording. For instance, Amx exposure resulted in a total distance of 7.68 ± 1.11 cm compared to a healthy control group, which covered a distance of 16.38 ± 1.17 cm. However, when the larvae were co‐exposed to L‐Mel and Amx, there was a potential improvement in locomotion. The combination of Amx and L‐Mel showed a total distance of 13.54 ± 0.63 cm. A graphical representation of the total distance travelled by the larvae is shown in (Figure 6D,E). Consistent with the findings from the larval behavioural assay, adult zebrafish exposed to amoxicillin (Amx) demonstrated a significant reduction in the total distance travelled relative to the controls (*p* < 0.0001). Notably, co‐administration with melatonin (Amx/Mel) or liposome‐encapsulated melatonin (Amx/L‐Mel) effectively mitigated Amx‐induced locomotor deficits, resulting in a significant restoration of activity (*p* = 0.0009 and *p* < 0.0001, respectively) (Figure 6F,G). Furthermore, a Python (v3.12.5)‐based pipeline (OpenCV, Matplotlib) was used to generate heatmaps, enabling group‐wise comparisons of treatment‐specific effects on locomotor and exploratory behaviours (Figure S3A).

### 3.6 Antioxidant and Proinflammatory Cytokine Assays for L‐Mel

**FIGURE 7:**
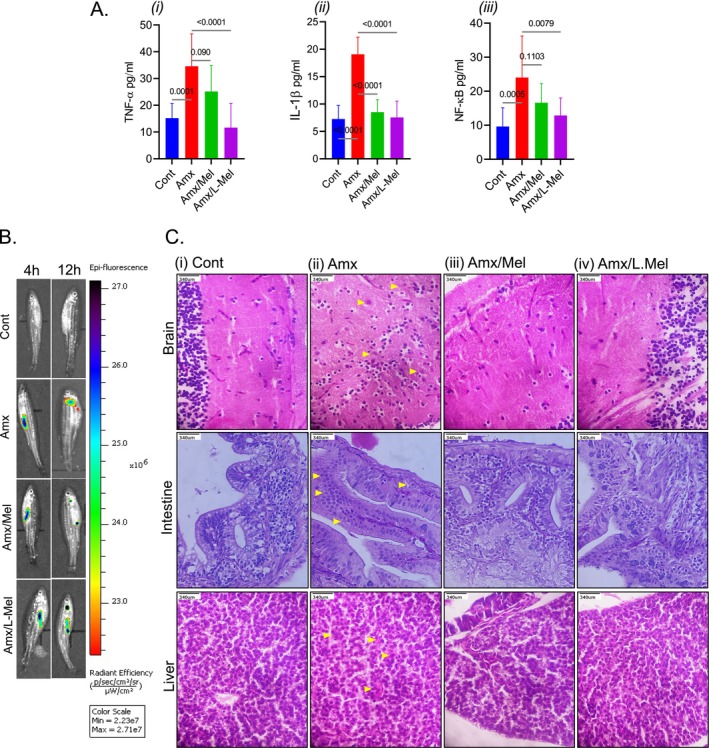
Effect of L‐Mel on proinflammatory cytokine (TNF‐α, IL‐1β, NF‐κB) levels in the head region of zebrafish larvae (*n* = 10 per group) (A). IVIS imaging of adult zebrafish (*n* = 10 per group) (B). Histopathology of adult zebrafish (*n* = 10 per group) brain, liver, and gut after exposure to Amx with and without Mel/L‐Mel (C). Statistical significance between the Amx‐treated and Amx/L‐Mel‐treated groups was set at *p < 0.05, **p < 0.01, ***p < 0.001. (*p < 0.05, **p < 0.01, and ***p < 0.001).

The enzyme levels of CAT (Figure 6H, i), GST (Figure 6H, v), and GSH (Figure 6H, vi) were significantly increased (*p* < 0.001) in the Amx/L‐Mel group compared to those in the Amx group. The GTPx activity (Figure 6H, iv) was notably increased (*p* < 0.05) in the Amx/L‐Mel group compared to that in the Amx group. Furthermore, in the case of SOD (Figure 6H, ii) and NO (Figure 6H, iii), there was a significant downregulation (*p* < 0.001) in the Amx/L‐Mel group compared to that in the Amx group. Moreover, in proinflammatory cytokines, the levels of TNF‐α [Amx (35.17 ± 1.04), Amx/L‐Mel (11.59 ± 1.08)], IL‐1β [Amx (18.70 ± 1.38), Amx/L‐Mel (7.18 ± 0.56)], and the NF‐κB levels were substantially decreased in the Amx/L‐Mel group (6.46 ± 0.67) compared to the Amx group (24.57 ± 0.71) (*p* < 0.001) (Figure 7A, i–iii).

### 3.7 IVIS Imaging and Histopathological Examination of Adult Zebrafish Treated With L‐Mel

In vivo imaging of zebrafish orally administered amoxicillin (Amx)‐rhodamine and a combination of Amx/Mel and Amx/L‐Mel‐rhodamine was used to track the action of the nano‐formulated drug (L‐Mel) in the zebrafish brain (Figure 7B). At 4 h postinjection, fluorescence intensity was observed in the peritoneal region of the zebrafish in the Amx, Amx/Mel, and Amx/L‐Mel groups. At 12 h postinjection, fluorescence was observed only in the head region of Amx‐administered fish and in both the head and peritoneal regions of Amx/Mel and Amx/L‐Mel‐administered fish. Interestingly, in the Amx/Mel group, the fluorescent emission was lower than that in the Amx/L‐Mel group, indicating that the nanoformulated drug (L‐Mel) persisted for a longer period in the circulation than raw Mel. This indicates that L‐Mel persists in the circulation along with Amx to ameliorate the damage caused by Amx in the brain. However, the histopathological variations were observed in three different organs: adult zebrafish's brain, liver, and intestine exposed to Amx and Amx/L‐Mel. Compared to the control group, the groups exposed to Amx exhibited tissue damage in all three organs, particularly in the brain. Conversely, fish administered Amx/L‐Mel showed less tissue damage, particularly in the brain, than in other organs. A significant change was observed in necrosis (Figure 7C, i–iv).

### 3.8 Gene Expression Pattern and HPLC Analysis of Adult Zebrafish Treated With L‐Mel

**FIGURE 8:**
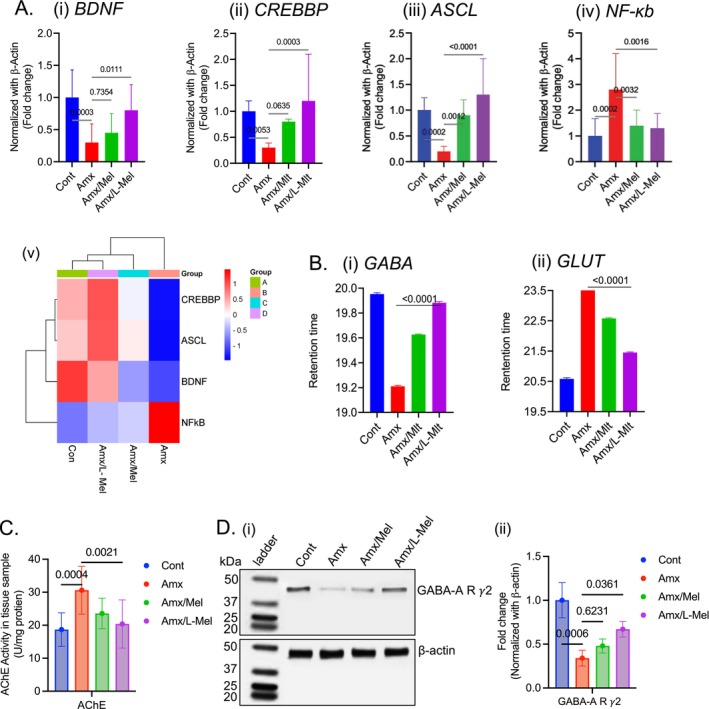
Gene expression in the brains of zebrafish larvae (*n* = 10 per group) was quantified using real‐time PCR (A, i–iv). A heat map was plotted for the candidate genes using the SR‐Plot software (A, v). Graphical representation of neurotransmitter levels in the adult zebrafish brain (*n* = 10 per group) (B i, ii). Statistical significance between the Amx‐treated and Amx/L‐Mel‐treated groups was set at *p < 0.05, **p < 0.01, ***p < 0.001. Acetylcholinesterase (AChE) activity in adult zebrafish brains (*n* = 10) showed elevated levels in the Amx group and reduced activity toward baseline in the Amx/L‐Mel group, indicating a protective modulatory effect of Amx/L‐Mel (C). Representation of western blot showing GABA‐A R γ2 expression levels under different conditions in adult zebrafish brain (*n* = 10 per group) (Amx, Amx/Mel and Amx/L‐Mel) (D, i). Graphical representation of the GABA‐A R γ2 expression level (D, ii). (*p < 0.05, **p < 0.01, and ***p < 0.001).

Melt curve analysis was performed, and the melt curve plot is shown in (Figure S4A). The amplification plot was plotted according to the fold‐change (Figure S4B). Interestingly, the BDNF level was notably higher (*p* < 0.05) in the Amx/L‐Mel group than in the Amx group. In addition, there was a notable increase (*p* < 0.01) in CREBBP and ASCL levels in the Amx/L‐Mel group compared to those in the group exposed to Amx alone. In contrast to the genes mentioned previously, the levels of NF‐κB were notably decreased (*p* < 0.01) in the Amx/L‐Mel group compared to those in the Amx group, as shown in (Figure 8A, i–iv). Moreover, with the gene expression data, a heat map was generated for the candidate genes using SR‐Plot, indicating the bidirectional gene cluster; for a clear understanding, this heat map illustrates the Euclidean distance of the targeted‐gene expression pattern. Blue represents lower values, whereas red represents higher values, as shown in (Figure 8A, v). Furthermore, in the HPLC analysis, the Amx group showed a decreased retention time of GABA (Rt‐Amx: 19.63) and increased retention time of glutamate (Rt‐Amx: 22.58) compared to the control (Rt‐GABA: 19.96, Rt‐Glutamate: 20.58) group, while Amx/L‐Mel showed a significantly increased retention time of GABA [Rt (Amx/L‐Mel): 19.89] and decreased retention time of glutamate [Rt (Amx/L‐Mel): 21.45] compared to the Amx group, as depicted in (Figure 8B, i–ii), and the results of HPLC (Figure S5A, i–iv).

### 3.9 Modulation of Acetylcholinesterase Activity in Adult Zebrafish Brains by Amx/L‐Mel Treatment

Furthermore, acetylcholinesterase (AChE) activity in adult zebrafish brains (*n* = 10) revealed that the AMX group exhibited a marked elevation compared to the control group, indicating enhanced cholinergic turnover. In contrast, the Amx/L‐Mel group demonstrated a pronounced reduction toward baseline levels, suggesting a protective role in maintaining the cholinergic balance (Figure 8C). The Amx/Mel group also showed an upregulation in AChE activity relative to the control. Collectively, these findings indicate that Amx/L‐Mel treatment exerts a favourable modulatory effect on cholinergic function in adult zebrafish.

### 3.10 L‐Mel Mitigate the Amx Induced—Neurotoxic Effect by GABA‐A R γ2 Protein Analysis in the Adult Zebra Fish Brain Tissue

Western blot analysis was performed to assess the expression levels of the target protein (GABA‐A R γ2) across the experimental samples (Control, Amx, Mel, and L‐Mel) (Figure 8D, i). Densitometric analysis revealed that the control group exhibited the highest expression compared to the Amx group. Further, Amx/L‐Mel group shows significant increase in the expression of the GABA‐A R γ2 in contrast to Amx and Amx/Mel conditions (*p* < 0.05) (Figure 8D, ii). The histogram shows these findings, with the control condition showing approximately 2‐fold higher expression levels than the Amx condition and 1.5‐fold higher than the Amx/Mel and Amx/L‐Mel conditions. The Figure S5B illustrates the complete Western blot showing GABA‐A R γ2 expression along with β‐actin.

## 4 Discussion

Amoxicillin is frequently prescribed because of its high susceptibility to bacterial infections. Adverse neurological effects have been associated with Amx treatment [51, 52]. A constrained study was conducted to assess the environmental impact of amoxicillin. This is likely because these substances have not been widely recognised as highly toxic based on the existing scientific literature [53, 54]. According to a study by Park and Choi [55], the 96‐h LC50 values for Amx were reported as 110.1 mg/L, respectively, for the fish species *Oryzias latipes* . In this study, embryos exposed to Amx for a short duration demonstrated premature hatching, consistent with findings reported by Oliveira et al. [7]. Amx neurotoxicity is primarily associated with the lactam ring, its capacity to penetrate the blood–brain barrier (BBB), and its connection to GABA receptors [56, 57].

In the present study, we explored the connection between Amx and neurological issues, with or without Mel treatment. According to a study conducted by Qiyao, Mel plays a major role in enhancing GABA receptor activity [14]. Moreover, using computational methods, we confirmed that Amx binds to the hydrogen bonds of ASP (A:473) and GLU (A:462) at multiple sites of GABA, and Mel specifically interacts with the hydrogen bond of MET (A:373) of the GABA receptor, revealing the targeted efficiency of Mel and Amx in the GABA receptor. Because of the short‐lasting periods of free Mel in the biological system, we identified a method to encapsulate Mel in a liposome‐mediated nanoformulation to improve its stability and sustained‐release compatibility in the biological system. Moreover, we stabilised and extended the efficacy of nanoformulated Mel (liposome‐encapsulated melatonin) to mitigate antibiotic‐induced neurological issues [58]. Melatonin acquired for the experiment was loaded into nanovesicles composed of PCDA and DMPC in a 4:1 ratio [21]. UV‐Spec, DLS, TEM, and FTIR spectroscopy were used to confirm the quality, size, and chemical characteristics of the formulated L‐Mel. Furthermore, XPS analysis revealed that L‐Mel is predominantly composed of sp^2^‐hybridised carbon integrated with conjugated C=O and C=N functionalities, together with oxygenated moieties. This heterogeneous surface chemistry is expected to impart enhanced structural stability and tunable reactivity, thereby broadening its potential for functional application. The resultant end product (L‐Mel) revealed that the nanoformulation was ideal for drug delivery.

Previously Jiang et al. [59] study reported that the antibiotic piperacillin caused mitochondrial malfunction and oxidative damage, leading to neuronal death. Similarly, our data corroborate that L‐Mel promotes viability and restores mitochondrial damage in an Amx‐induced in vitro neuroinflammation model. In accordance with Jiang et al., ROS and mitochondrial superoxide assays were used to detect mitochondrial dysfunction in zebrafish larvae. However, our results also demonstrate that L‐Mel can inhibit developmental abnormalities and improve behavioural movements. In another study by Gonçalves et al. [60, 61], high exposure to Amx altered antioxidant levels in a young zebrafish model. However, in our study, L‐Mel successfully inhibited high exposure to Amx‐induced neuronal damage in zebrafish larvae and significantly restored the antioxidant levels of CAT, GST, GSH, GTPx, SOD, and NO [62, 63].

A recent study demonstrated that TNF‐α and TNF‐β trigger apoptosis of head leukocytes in Nile tilapia [64]. Based on a previous report, we examined the involvement of TNF‐α, IL‐1β, and NF‐κB as proinflammatory cytokines in zebrafish and demonstrated that L. Mel significantly reduced Amx‐induced apoptosis. According to our histopathological results, the group treated with Amx experienced significant cell damage in the brain tissue compared to that in the Amx/L‐Mel group [65]. Based on gene expression analysis, genes linked to memory and behaviour were selected, including BDNF, which plays a crucial role in the survival and growth of neurons, acts as a modulator of neurotransmitters, and contributes to neuronal plasticity, a vital component of memory and cognition processes [66]. CREBBP is crucial for regulating cell division and proliferation and inducing cells to mature and perform specific functions [67, 68]. ASCL is a key regulator of neurogenesis in the mammalian brain. NF‐κB ensures the well‐being of neurons, promotes the growth of synapses, and supports functions related to plasticity [69, 70]. Our research revealed that L‐Mel regulates the expression of these genes and protects the brain from neuronal injury. Based on the literature, an increase in glutamate, an excitatory amino acid, is strongly linked to the development of neurological disorders [32, 71, 72]. Moreover, we demonstrated a significant upregulation of GABA levels and a corresponding downregulation of glutamate levels in L‐Mel‐treated zebrafish brain tissue. In addition, the protein expression of GABA‐A R γ2 revealed that L‐Mel counteracts Amx‐induced neuroinflammation and exerts long‐lasting effects within the biological system. The observed reduction in acetylcholinesterase (AChE) activity in the Amx/L‐Mel group further suggests a protective role in maintaining cholinergic signalling and preventing excessive acetylcholine breakdown. Collectively, these findings highlight the neuroprotective potential of Amx/L‐Mel through the modulation of neurotransmitter balance, attenuation of neuroinflammation, and preservation of cholinergic function in adult zebrafish.

## 5 Conclusion

In summary, PCDA‐DMPC liposome‐mediated Mel promoted efficiency and adequately demonstrated sustained drug release under ex vivo conditions. Computational analysis confirmed the binding affinities of Amx and Mel for the GABA protein model. L‐Mel efficiently inhibits the abnormalities caused by Amx and regulates behavioural and antioxidant levels in both in vitro and in vivo models. Moreover, L‐Mel regulates neurotransmitter levels and mitigates neuronal degeneration during AMX‐induced brain injuries. Therefore, our study demonstrates that nanoformulated L‐Mel is a promising molecule for addressing antibiotic‐induced neurological issues. However, further preclinical research is required to validate the extended therapeutic advantages of L‐Mel in treating neuroinflammation and antibiotic‐induced behavioural impairment.

## Author Contributions

**Ranjith Balakrishnan:** conceptualization (equal), formal analysis (equal), investigation (equal), methodology (equal), software (equal), validation (equal), visualization (equal), writing – original draft (equal). **Rajasekaran Subbarayan:** conceptualization (equal), formal analysis (equal), investigation (equal), methodology (equal), resources (equal), supervision (equal), validation (equal), visualization (equal), writing – original draft (equal). **Rupendra Shrestha:** conceptualization (equal), formal analysis (equal), investigation (equal), resources (equal), validation (equal), visualization (equal), writing – review and editing (equal). **Dhasarathdev Srinivasan:** formal analysis (equal), investigation (equal), methodology (equal), software (equal), validation (equal), visualization (equal). **Reena Shrestha:** formal analysis (equal), validation (equal), writing – review and editing (equal). **Ankush Chauhan:** formal analysis (equal), validation (equal), writing – review and editing (equal). **Dinesh Murugan Girija:** formal analysis (equal), validation (equal), writing – review and editing (equal).

## Funding

This study was self‐funded and did not receive any external financial support.

## Ethics Statement

The study was approved by the Animal Ethics Committee of the Chettinad Hospital and Research Institute (CHRI), Chettinad Academy of Research and Education, India.

## Conflicts of Interest

The authors declare no conflicts of interest.

## Supporting information


**Figure S1:** SAS interactions and aromatic bonding between the protein (GABA) and ligands (Amx and Mel) (A and B).
**Figure S2:** Ramachandran plot analysis indicated that a significant majority of residues (> 80.0%) were situated in the favourable region of Amx and Mel when interacting with GABA. This observation is demonstrated through the Psi and Residue Index (A and B).
**Figure S3:** Representative heatmaps depicting the locomotor activity of adult zebrafish across the treatment groups: (i) Con, (ii) Amx, (iii) Amx/Mel, and (iv) Amx/L‐Mel. Colour intensity denotes the frequency of spatial occupancy, highlighting treatment‐dependent alterations in exploratory behaviour (A).
**Figure S4:** Representation of the melt curve analysis plot (A). An amplification plot was generated according to the fold change (B).
**Figure S5:** HPLC data representing the retention time of the neurotransmitters (GABA & Glutamate) (A: I‐iv). Representation of uncropped western blot showing protein expression levels with loading control (B).

## Data Availability

The data supporting the findings of this study are available in the Supporting Information section of this article.
