# Supplementary material for: Liposome‐Encapsulated Melatonin Mitigates Amoxicillin‐Induced Neurotoxicity in a Zebrafish
Source: J Cell Mol Med. 2025 Nov 26;29(22):e70969. doi: 10.1111/jcmm.70969 (PMC12648294; doi:10.1111/jcmm.70969)
Supplement: Supplementary file 1 — Figure S1: SAS interactions and aromatic bonding between the protein (GABA) and ligands (Amx and Mel) (A and B). Figure S2: Ramachandran plot analysis indicated that a significant majority of residues (> 80.0%) were situated in the favourable region of Amx and Mel when interacting with GABA. This observation is demonstrated through the Psi and Residue Index (A and B). Figure S3: Representative heatmaps depicting the locomotor activity of adult zebrafish across the treatment groups: (i) Con, (ii) Amx, (iii) Amx/Mel, and (iv) Amx/L‐Mel. Colour intensity denotes the frequency of spatial occupancy, highlighting treatment‐dependent alterations in exploratory behaviour (A). Figure S4: Representation of the melt curve analysis plot (A). An amplification plot was generated according to the fold change (B). Figure S5: HPLC data representing the retention time of the neurotransmitters (GABA & Glutamate) (A: I‐iv). Representation of uncropped western blot showing protein expression levels with loading control (B). [file JCMM-29-e70969-s001.docx]

**Supplementary Data**

**Supplementary Figures**

**
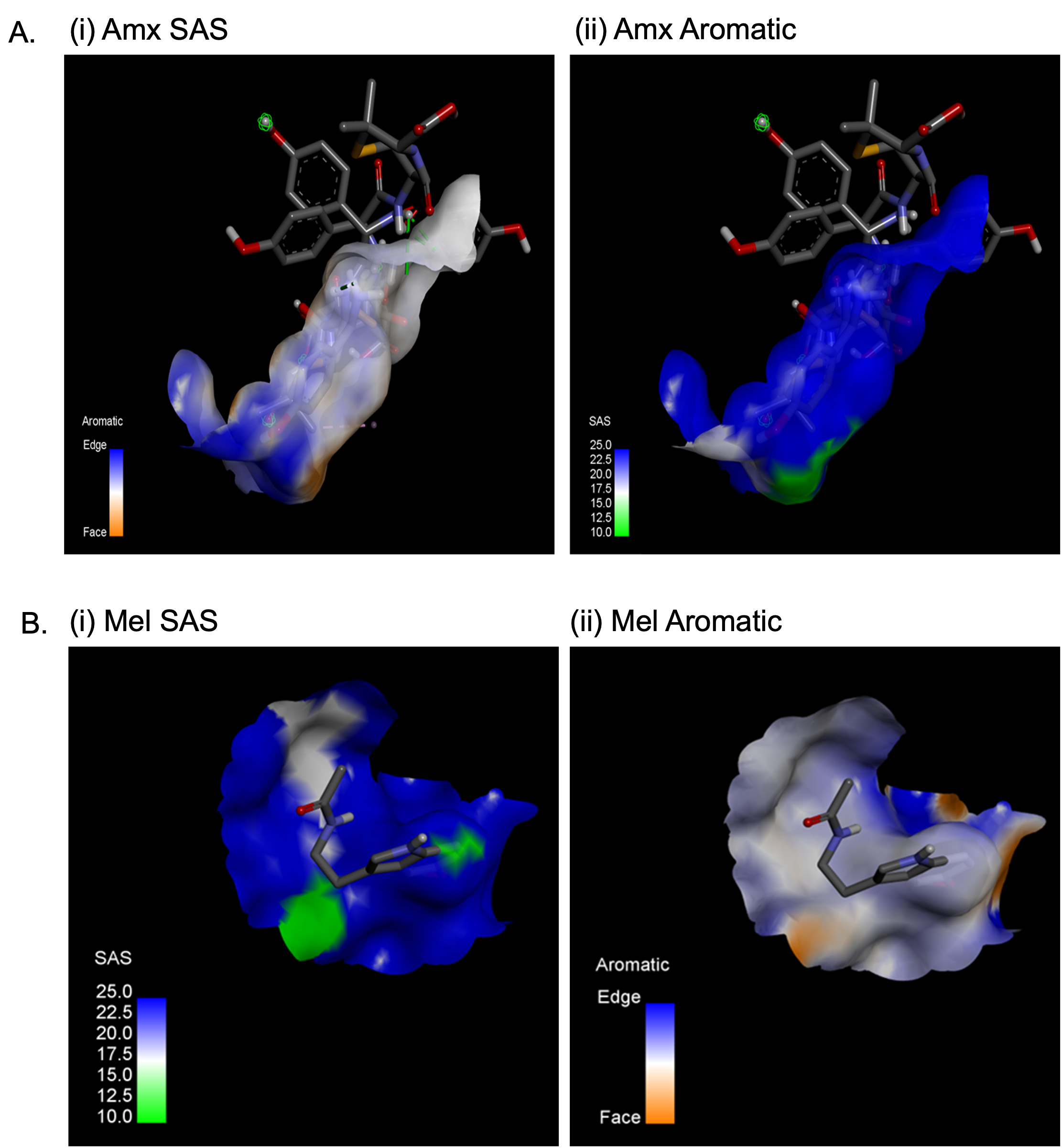
**

**Supplementary Figure 1:** SAS interactions and aromatic bonding between the protein (GABA) and ligands (Amx and Mel) (A and B).

**
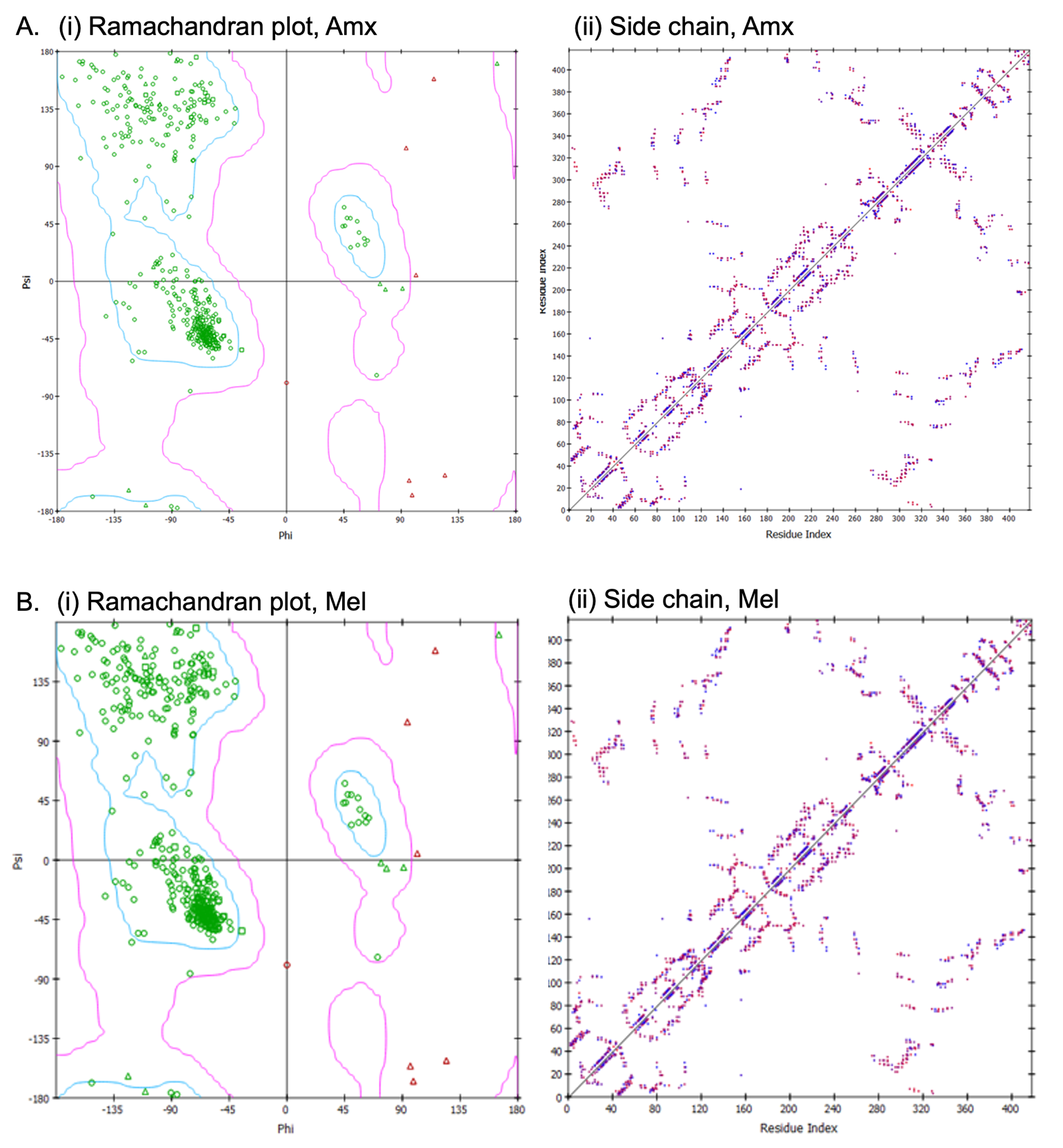
**

**Supplementary Figure 2:** Ramachandran plot analysis indicated that a significant majority of residues (> 80.0%) were situated in the favorable region of Amx and Mel when interacting with GABA. This observation is demonstrated through the Psi and Residue Index (A and B).


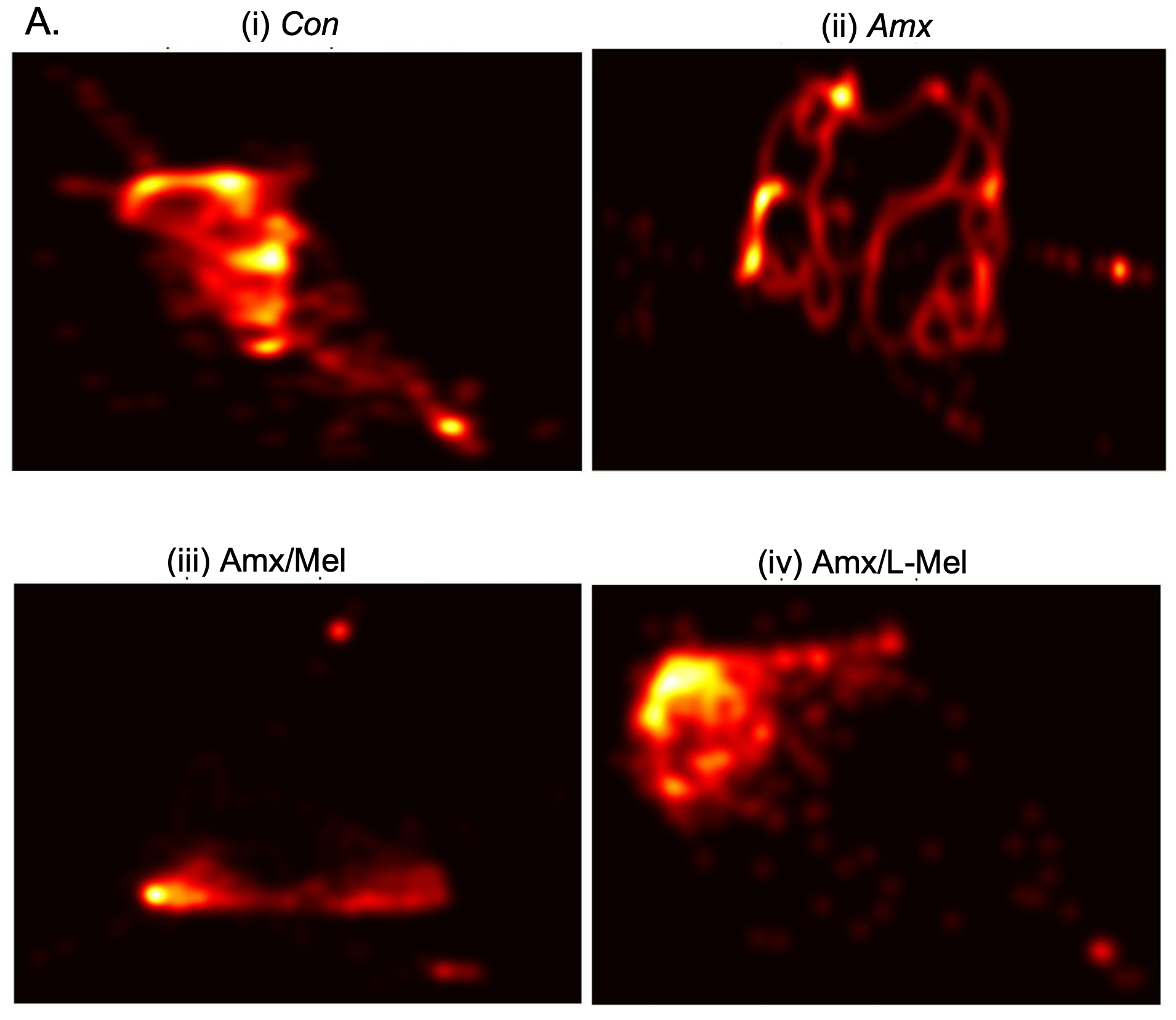


**Supplementary Figure 3:** Representative heatmaps depicting the locomotor activity of adult zebrafish across the treatment groups: (i) Con, (ii) Amx, (iii) Amx/Mel, and (iv) Amx/L-Mel. Color intensity denotes the frequency of spatial occupancy, highlighting treatment-dependent alterations in exploratory behavior (A).


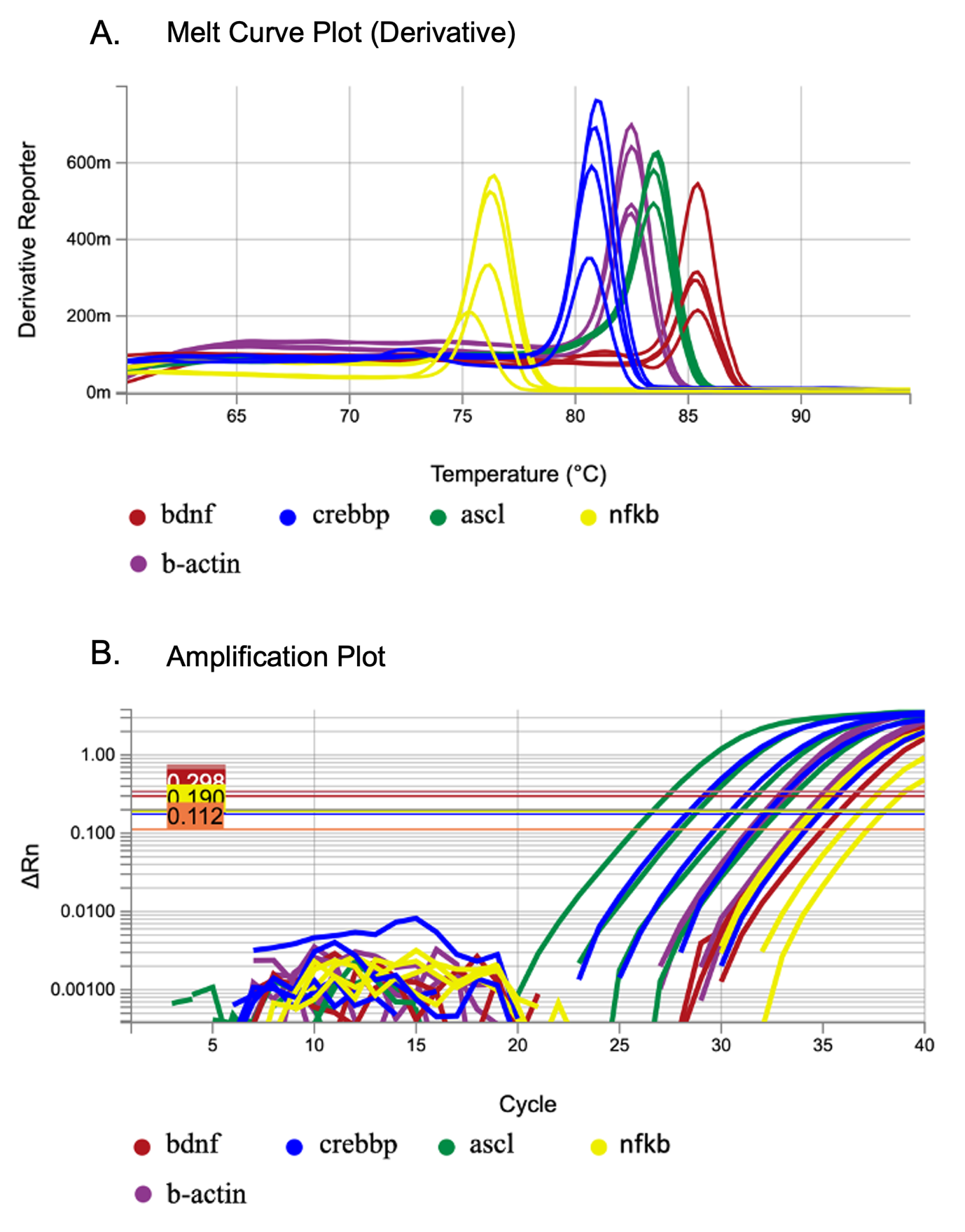


**Supplementary Figure 4:** Representation of the melt curve analysis plot (A). An amplification plot was generated according to the fold change (B).


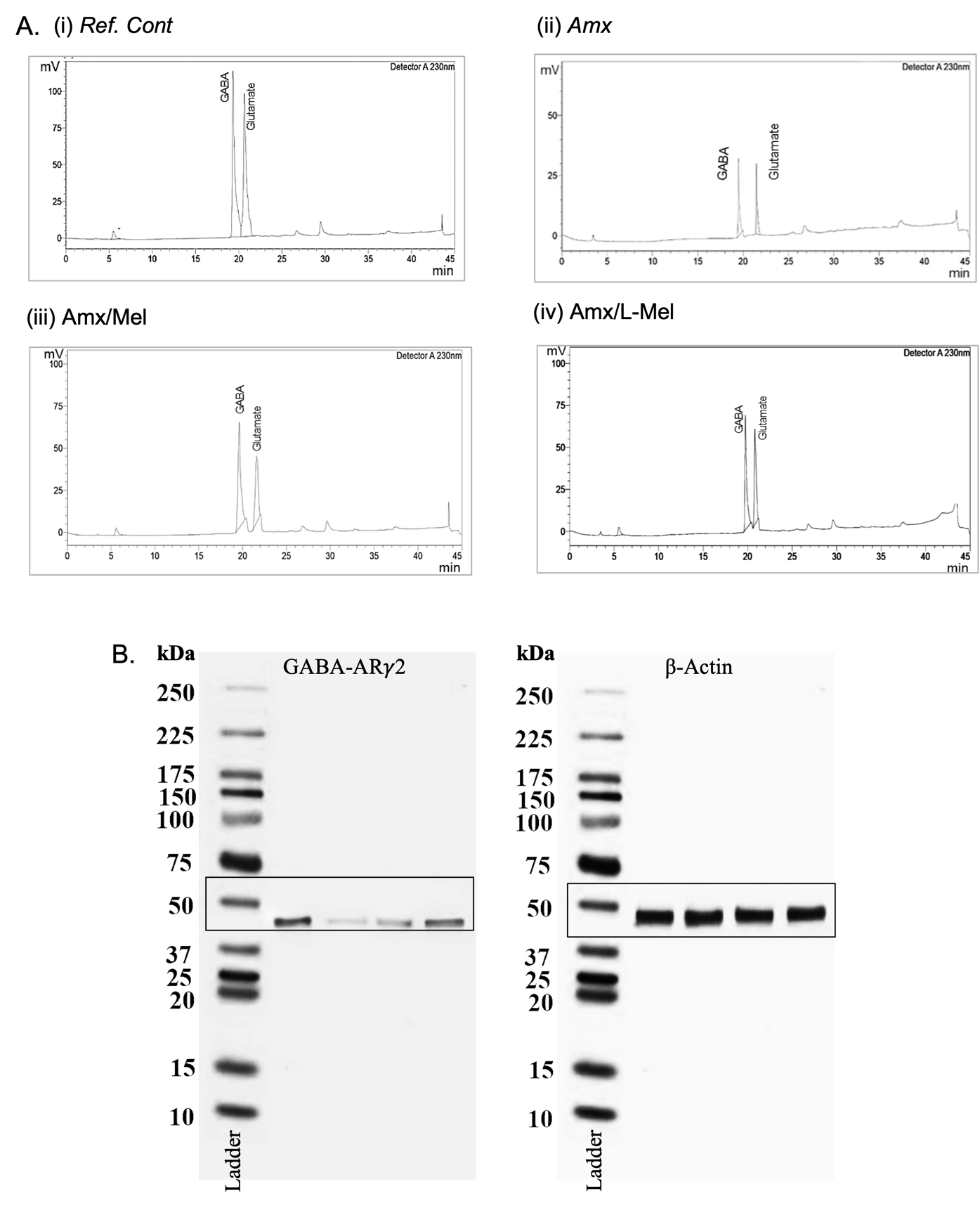
**Supplementary Figure 5:** HPLC data representing the retention time of the neurotransmitters (GABA & Glutamate) (A: i-iv). Representation of uncropped western blot showing protein expression levels with loading control (B).
